# Dose‐dependent selection drives lineage replacement during the experimental evolution of SDHI fungicide resistance in *Zymoseptoria tritici*

**DOI:** 10.1111/eva.12511

**Published:** 2017-09-03

**Authors:** Omar Gutiérrez‐Alonso, Nichola J. Hawkins, Hans J. Cools, Michael W. Shaw, Bart A. Fraaije

**Affiliations:** ^1^ Biointeractions and Crop Protection Department Rothamsted Research Harpenden Hertfordshir UK; ^2^ Jealott's Hill International Research Centre Syngenta Bracknell Berkshire UK; ^3^ School of Agriculture Policy and Development University of Reading Reading Berkshire UK

**Keywords:** adaptation, agriculture, development and evolution, experimental evolution, microbial biology, molecular evolution, natural selection and contemporary evolution

## Abstract

Fungicide resistance is a constant threat to agricultural production worldwide. Molecular mechanisms of fungicide resistance have been studied extensively in the wheat pathogen *Zymoseptoria tritici*. However, less is known about the evolutionary processes driving resistance development. In vitro evolutionary studies give the opportunity to investigate this. Here, we examine the adaptation of *Z. tritici* to fluxapyroxad, a succinate dehydrogenase (Sdh) inhibitor. Replicate populations of *Z. tritici* derived from the sensitive isolate IPO323 were exposed to increasing concentrations of fluxapyroxad with or without UV mutagenesis. After ten increases in fungicide concentration, sensitivity had decreased dramatically, with replicate populations showing similar phenotypic trajectories. Sequencing the Sdh subunit B, C, and D encoding genes identified seven mutations associated with resistance to fluxapyroxad. Mutation frequency over time was measured with a pyrosequencing assay, revealing sequential lineage replacement in the UV‐mutagenized populations but not in the untreated populations. Repeating selection from set time‐points with different fungicide concentrations revealed that haplotype replacement of Sdh variants was driven by dose‐dependent selection as fungicide concentration changed, and was not mutation‐limited. These findings suggest that fungicide field applications may select for highly insensitive Sdh variants with higher resistance factors if the fungicide concentration is increased to achieve a better disease control. However, in the absence or presence of lower fungicide concentrations, the spread of these strains might be restricted if the underlying *Sdh* mutations carry fitness penalties.

## 1 INTRODUCTION

Populations require genetic variability to adapt to new environments or toxicants. As well as natural standing variation (Barrett & Schluter, 2008; Hawkins et al., 2014), genetic variability can arise from de novo mutations (Lenski, 2004; Torriani, Brunner, McDonald, & Sierotzki, 2009). The mutation frequency and the order in which beneficial mutations accumulate can drive the evolutionary adaptation of pathogens to toxicants (Cowen et al., 2000; Weinreich, Delaney, Depristo, & Hartl, 2006). Laboratory evolution studies give the opportunity to investigate the process of adaptation to new selective pressures including toxicants. Lenski, Rose, Simpson, and Tadler (1991) founded a long‐term evolution experiment from a single *Escherichia coli* bacterium in 1988 to characterize the dynamics of adaptive evolution over long‐term periods under a constant environment. Since then, 12 replicate *E. coli*‐derived populations have been evolving for over 60,000 generations (Cooper, Rozen, & Lenski, 2003; Cooper, Schneider, Blot, & Lenski, 2001; Lenski & Travisano, 1994; Woods, Schneider, Winkworth, Riley, & Lenski, 2006). Keeping a “fossil record” in the form of glycerol stocks of intermediate generations of the *E. coli*‐derived populations allowed the experimental evolution to be repeated from specific time‐points. This enabled investigation into the repeatability of evolution under different conditions and the role of chance mutations (Blount, Borland, & Lenski, 2008).

Studies examining the evolution of resistance to toxicants have found parallel increases in resistance between replicate populations. Cowen et al. (2002) reported parallel evolution of resistance to fluconazole after 330 generations in four replicate populations of *Candida albicans*. Resistance was conferred by overexpression of either an ATP‐binding cassette (ABC) transporter or a multidrug major facilitator (MFS) transporter encoding gene. Similarly, resistance to fluconazole in *Saccharomyces cerevisiae* was also found to be conferred by overexpression of two ABC transporters (*pdr5* or *snq2*) after 400 generations in replicate populations subjected to increasing concentrations of the fungicide (Anderson et al., 2003). Toprak et al. (2012) also found parallel development of resistance in 12 replicate populations of *E. coli* after approximately 20 days under an increasing concentration of antibiotics. Resistance was conferred by combinations of mutations in three distinct genes or by accumulation of mutations in one single gene. However, none of the previous studies provided insights into the evolutionary process of adaptation to fungicides. This study extends this experimental evolutionary approach to investigate the selection process of resistance to a new class of fungicides in a plant pathogen.

*Zymoseptoria tritici*, the causal agent of Septoria leaf blotch (SLB) in wheat, is a highly adaptable fungal pathogen (Zhan & McDonald, 2004). This plant pathogen has developed resistance or reduced sensitivity to fungicides with different mode of action, including methyl‐benzimidazole carbamates (MBCs) (Griffin & Fisher, 1985), quinone outside inhibitors (QoIs) (Amand et al., 2003; Fraaije et al., 2005), and sterol‐demethylation inhibitors (DMIs) (Clark, 2006; Cools & Fraaije, 2013). Therefore, options for the chemical control of SLB are currently limited. Since 2003, a new generation of succinate dehydrogenase inhibitor (SDHI) fungicides (e.g., boscalid, isopyrazam, bixafen, penthiopyrad, and fluxapyroxad) with strong activity against SLB has been launched in the crop protection market (Fraaije et al., 2012; Scalliet et al., 2012). SDHI fungicides target the protein succinate dehydrogenase (Sdh), also known as succinate ubiquinone oxidoreductase, that consists of four subunits. SdhA oxidises succinate to fumarate as a component of the tricarboxylic acid cycle (TCA), which is coupled with the reduction of ubiquinone to ubiquinol as part of the mitochondrial electron transport chain (Iverson, 2013). The SDHIs exert their fungicidal action by physically blocking the ubiquinone‐binding pocket, formed by Sdh subunits B, C, and D (Ulrich & Mathre, 1972).

Laboratory studies using single generation exposures to different SDHIs have reported several target‐site mutations conferring reduced sensitivity in mutants of *Z. tritici* and other plant pathogens (Fraaije et al., 2012; Scalliet et al., 2012; Sierotzki & Scalliet, 2013; Skinner et al., 1998). The SDHI sensitivity can be differentially affected by mutations. For example, SdhB‐H267Y mutants of *Z. tritici* are insensitive to boscalid but hypersensitive to fluopyram. Other mutations of laboratory mutants, such as SdhB‐H267L, SdhC‐N86K, and SdhD‐D129G, confer high levels of resistance to all SDHIs, including fluopyram. Due to the resistance risk, the SDHIs are used in mixtures with azoles or multisite inhibitors at recommended rates to delay fungicide resistance development in *Z. tritici* (FRAC, 2015). Field monitoring studies conducted since 2003 first detected sensitivity changes in 2012. Between 2012 and 2015, five different Sdh variants, SdhB‐N225T, B‐T268I, C‐T79N, C‐W80S, and C‐N86S, were reported at low frequencies in France, Germany, Ireland and the UK (FRAC, 2016). These Sdh variants display low levels of insensitivity, and control of SLB has not been affected so far. However, this may change as field strains carrying C‐H152R, showing high resistance factors to SDHIs in vitro, have recently been detected in Ireland (Dooley, Shaw, Mehenni‐Ciz, Spink, & Kildea, 2016) and the UK (B. A. Fraaije, unpublished), albeit at low levels.

Insensitivity to SDHIs has evolved in *Z. tritici* field populations as predicted from the variants found in laboratory mutational experiments. However, the extent of fitness penalties associated with mutations in the target protein conferring resistance to SDHIs, and the functional constrains affecting Sdh evolution under SDHI field selection where populations are exposed to different dose rates and spray frequencies remain unknown. In vitro evolutionary studies enabling to accelerate the evolution give the opportunity to investigate this.

In this study, we determined the course of evolution of resistance to the Sdh inhibitor fluxapyroxad in replicate populations of *Z. tritici* generated from the SDHI sensitive reference isolate IPO323 (Kema & van Silfhout, 1997). The IPO323 derived populations were exposed to increasing inhibitor concentrations in replicate populations at three different starting concentrations, each with or without exposure to UV light to increase mutation rate. After adaptation to ten stepwise increases in fungicide concentration, mutants carrying different *Sdh* mutations were found in most populations. One population without exposure to UV showed relatively low levels of SDHI insensitivity in the absence of target‐site mutations. Studies on archived populations over time showed the mutation rate was not a limiting factor in UV‐mutagenized lines and that the haplotype replacement of Sdh variants was governed by dose‐dependent selection as the “selective window” of the fungicide concentration changed.

## 2 MATERIAL AND METHODS

### 2.1 Generation of fluxapyroxad‐resistant mutants

The reference *Z. tritici* isolate IPO323 (Kema & van Silfhout, 1997) was used as the parental strain in all experiments. For this, aliquots of 100 μl of IPO323 spore suspensions (10^7^ spores/ml) were plated out onto 60‐mm petri dishes of YPD agar amended with 0.4% (v/v) DMSO, and 0.04, 0.06, or 0.08 μg/ml of fluxapyroxad formulated as an emulsifiable concentrate (62.5 g/L EC; BASF, Ludwigshafen, Germany). Four YPD plates per fluxapyroxad concentration were inoculated, of which two were then exposed to 300 J/m^2^ of UV light using an UV Crosslinker (model: XLE‐1000/FB; Spectroline, New York, NY, USA). The UV light exposure of 300 J/m^2^ allowed approximately 45% survival. After exposure, all cultures were incubated at 21°C in the dark for seven days.

After seven days' incubation, spores were harvested into sterile distilled water and quantified by haemocytometer count. Cultures with at least 10^7^ spores/ml were exposed to an increased concentration of fungicide. An aliquot of 100 μl of spore suspension (10^7^ spores/ml) from each culture was transferred to new YPD plates amended with 0.4% (v/v) DMSO and a twofold increased concentration of fluxapyroxad and left untreated or exposed to 300 J/m^2^ of UV light before further incubation. The remaining spore suspension of each culture at each transfer was suspended in 80% (v/v) glycerol and stored at 80°C for further studies. Cultures with less than 10^7^ spores/ml were plated on the same fluxapyroxad concentration and left in the dark at 21°C for another generation of seven days until they reached the spore concentration needed to be transferred on to a doubled fungicide concentration. Every series of fungal cultures was kept separately, as an independent population, labelled as “L”, “I,” or “H” for populations initially exposed to a low (0.04 μg/ml), intermediate (0.06 μg/ml), or high (0.08 μg/ml) concentration of fluxapyroxad, respectively, and each with (+) or without (−) UV exposure. Replicate populations can be distinguished by the label 1 or 2 added to each condition (i.e., L−1, L−2, and L+1, L+2; see Fig. S1).

After ten rounds of selection (RS) at increasing concentrations of fungicide (from 0.04 to 20.48 μg/ml, 0.06 to 30.72 μg/ml or 0.08 to 40.96 μg/ml), fluxapyroxad‐adapted mutants were isolated. Populations were diluted to produce single colonies on unamended YPD plates, and twenty colonies from each population were picked and subcultured on unamended YPD for seven days at 21°C in the dark, and stored in 80% glycerol (v/v) at −80°C for further use. Mutants were named in progressive number order per population from which they came (e.g., from L−1.1 to L−1.20).

### 2.2 Fungicide sensitivity testing

In vitro fungicide sensitivity assays were carried out as described by Pijls, Shaw, and Parker (1994), see Supporting information. After four days of fungicide exposure at 23°C in the dark, fungal growth was measured using absorbance readings at 630 nm (A_630_) with a FLUOstar OPTIMA microplate reader (BMG Labtech GmbH, Offenberg, Germany). Absorbance was measured in a well‐scanning mode with a 2 × 2 matrix of scanning points set at 3 mm diameter. Fungicide sensitivity was determined as the concentration, which inhibited growth by 50% (EC_50_). The EC_50_ (μg/ml) values were calculated using a dose–response curve (four‐parameter fit) using OPTIMA software (v2.20OR2). The fungicide sensitivity test was carried out twice, and the progenitor/reference isolate IPO323 was included as control. The EC_50_ values are the average of the two independent tests.

To determine variation among the populations, the EC_50_ values were analysed using ANOVA to compare the populations, taking account of initial fungicide concentration and UV light treatment, in GenStat (2014, 17th edition, VSN International^©^, Hemel Hempstead, UK). The standard error of the difference (SED) based on the residual degrees of freedom (*df*) from the ANOVA was used to construct a least significant difference (LSD) at the 5% level of significance with which to compare relevant means.

### 2.3 Sequencing of the Sdh subunit B, C, or D encoding genes

The binding site of SDHI fungicides is formed by Sdh subunits B, C, and D, and mutations conferring resistance in *Z. tritici* have been previously identified in all three subunits (Fraaije et al., 2012; Scalliet et al., 2012). To determine whether Sdh alterations were associated with SDHI insensitivity, two fluxapyroxad‐insensitive mutants with each of the highest, median, or lowest fluxapyroxad EC_50_ values were selected from each population and their *SdhB*,* C,* and *D* genes sequenced.

Genomic DNA for sequencing was extracted and quantified according Rudd et al. (2010) from strains grown on YPD plates at 15°C in the dark for seven days. PCRs were carried out on a Biometra T3000 thermocycler (Biotron, Göttingen, Germany) in a final volume of 50 μl containing 50 ng of fungal template DNA. PCRs for amplification of *SdhB* or *D* contained 0.5 μM for each primer (Table S1) and 200 μM dNTP, 1× Phusion HF buffer, and 1.0 units of Phusion High Fidelity DNA polymerase (New England Biolabs, Ipswich, MA, USA). Polymerase chain reactions for amplification of *SdhC* were 0.2 μM for each primer (Table S1) and 200 μM dNTP and contained 1× of Easy‐A reaction buffer and 2.5 units of Easy‐A High Fidelity PCR cloning enzyme (Agilent Technologies, Cedar Creek, TX, USA). Amplification conditions were 95°C for 1 min, followed by 30 cycles at 95°C for 15 s, 70°C (*SdhB* and *D*) or 65°C (*SdhC*) for 30 s, and 72°C for 1 min with a final DNA extension at 72°C for 5 min. PCR products were sequenced by MWG Eurofins Genomics GmbH (Ebersberg, Germany) using specific primers for each *SdhB*,* C,* or *D* gene (Table S1). Sequences were assembled and aligned with Geneious v.6.1.4 software (Biomatters Ltd., Auckland, New Zealand), and amino acid substitutions determined after sequence analysis.

### 2.4 SNP detection pyrosequencing assays

To determine changes in frequency of key DNA mutations linked with SDHI resistance at each round of selection, six populations: three exposed to UV and with a greater diversity of mutations (I+1, H+1, and H+2), and the others without UV exposure and with fewer or no mutations (L−1, I−1, and H+1) were tested using SNP detection pyrosequencing assays using the PyroMark Q96 system (Biotage, Uppsala, Sweden) as described by Carter, Cools, West, Shaw, and Fraaije (2013) (see Supporting information). Aliquots of 10 μl of spore suspension from the last round of selection, and stored glycerol spore suspensions of previous RS, were plated out on YPD agar, and incubated for seven days at 15°C in the dark. DNA was extracted from each population. Mutants carrying different Sdh variants and the reference isolate IPO323 were used as positive and negative controls for the SNP detection pyrosequencing assays. Frequencies of mutations underlying SdhB‐H267L, B‐H267Y, B‐N225T, C‐T79I, C‐S83G, C‐H152R, and D‐I50L were estimated with the PyroMark Q96 ID software version 2.5. Frequency values are the mean of two technical replicate pyrosequencing reactions. The detection limit of SNP detection pyrosequencing is approximately 3% for each allele (Gruber, Colligan, & Wolford, 2002; Wasson, Skolnick, Love‐Gregory, & Permutt, 2002).

### 2.5 Effect of fluxapyroxad concentration on the selection of SdhC‐T79I and C‐H152R variants

To investigate whether lineage replacement is mutation‐limited or due to alleles being favoured only at certain stages of the selection regime, population H+1 spore suspensions from RS 3, 4, or 7 were regrown on YPD plates for seven days at 15°C in the dark and subsequently exposed to different concentrations of fluxapyroxad. For this, aliquots of 100 μl of RS3, RS4, or RS7 spore suspensions at 10^7^ spores/ml were plated out onto YPD plates amended with 0.4% (v/v) DMSO, and 0, 0.32, 0.64, or 5.12 μg/ml of fluxapyroxad. These concentrations correspond to the most recent round of selection at which these populations were originally selected. Inoculated plates were incubated for seven days at 21°C in the dark, and DNA was extracted. Frequencies of SdhC‐T79I or C‐H152R variants in these populations were determined using SNP detection pyrosequencing assays.

### 2.6 Gene expression studies

Although mutants of population L−1 were less sensitive to fluxapyroxad, no target‐site mutations were found in the *SdhB*,* C,* or *D* gene. To investigate whether changes in gene expression of a range of nontarget‐site resistance conferring candidate genes were associated with fluxapyroxad insensitivity, we examined changes in their mRNA levels following the introduction of the fungicide. The reference *Z. tritici* isolate IPO323 and the fluxapyroxad‐adapted mutant L‐1.7, with no target‐site mutations in the *SdhB*,* C,* or *D* gene, were in vitro exposed to fluxapyroxad at concentrations inhibiting growth by 50 or 80% (EC_80_), and changes in gene expression was measured (see Supporting information).

Relative transcript abundances [relative quantities (RQ)] from genes encoding SdhB, C, or D, alternative oxidase (AOX), seven ABC transporters, seven major facilitator superfamily (MFS) drug efflux transporters, seven glutathione S‐transferase (GST), and β‐tubulin were determined with quantitative RT‐PCR using specific oligonucleotide primers (Table S3). The selected genes encoding transporters or GSTs were the seven most up‐regulated genes in the *Z. tritici* isolate IPO323 after exposure to chlorothalonil or folpet in the lag or log phase of growth (Gutierrez‐Alonso, 2015). The *Aox* gene was included because overexpression can confer lower sensitivity to other fungicides that affect respiration (QoI) through nontarget‐site mutations (Miguez, Reeve, Wood, & Hollomon, 2004).

Normalized relative quantities (NRQ) of target genes were calculated by the 2^−[Δ][Δ]*C*t^ method (Pfaffl, 2001), using β‐tubulin as the endogenous control and samples from the untreated IPO323 isolate as calibrator. NRQ values of selected target genes were transformed to the logarithmic (log_2_) scale and analysed using ANOVA in GenStat. The log_2_(NRQ) values were calculated assuming an efficiency of two for the target and reference gene (Pfaffl, 2001). Results are presented as ratios to the control treatment, using the log_2_ (NRQ) means.

## 3 RESULTS

### 3.1 Generation of fluxapyroxad‐resistant *Zymoseptoria tritici* mutants

Six fluxapyroxad‐adapted populations (L+1, L+2, I+1, I+2, H+1, and H+2) derived from the sensitive *Z. tritici* isolate IPO323 were obtained after ten RS at increasing fungicide concentrations and UV exposure (Fig. S1). Three fluxapyroxad‐adapted populations (L−1, I−1 and H−1) were obtained using the same series of fungicide concentrations but without UV light exposure (Fig. S1). The UV unexposed populations L−2, I−2, and H−2 stopped growing at week 11, 3, and 4, respectively (Fig. S1).

Population L−1 was obtained after 17 weeks, and populations L+1 and L+2 were obtained after 12 weeks, using fluxapyroxad concentrations from 0.04 to 20.48 μg/ml (Fig. S1A). Population I−1 was obtained after 18 weeks, and populations I+1 and I+2 were obtained after 12 weeks, using fluxapyroxad concentrations from 0.06 to 30.72 μg/ml (Fig. S1B). Populations H−1, H+1, and H+2 were obtained after 12 weeks, using fluxapyroxad concentrations from 0.08 to 40.96 μg/ml (Fig. S1C). A total of 180 fluxapyroxad‐adapted IPO323 mutants were isolated, 20 from each final population.

### 3.2 Sensitivity of fluxapyroxad‐resistant mutants to SDHIs

**Figure 1:**
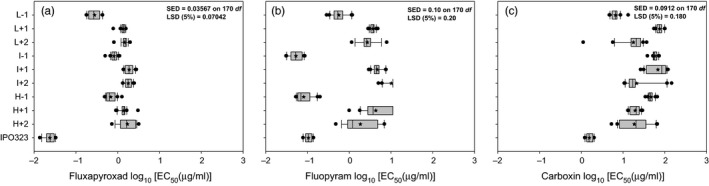
Sensitivities to fluxapyroxad (a), fluopyram (b), and carboxin (c) of *Zymoseptoria* *tritici* IPO323‐derived populations after ten increases of fungicide on YPD amended with 0.04 (L−1, L+1, L+2), 0.06 (I−1, I+1, I+2), or 0.08 (H−1, H+1, H+2) μg/ml of fluxapyroxad as starting concentration. Fungicide concentration was increased twofold every round of selection. Populations L+1, L + 2, I + 1, I + 2, H + 1, and H + 2 were also exposed to 300 J/m^2^ of UV light. Sensitivity values are the mean of two independent experiments. Star indicates the mean of each population consisting of 20 isolates. Closed circles indicate values beyond the 10th and 90th percentiles. Sensitivity of the parent IPO323 isolate is the average of ten technical replicates

Isolates from the fungicide‐adapted populations were less sensitive to fluxapyroxad than the reference isolate IPO323, which had an average EC_50_ value of 0.02 μg/ml in this study (Figure 1a; Table S4). No significant differences (*p *=* *.2) were detected between EC_50_ values of the nine populations studied. Fluxapyroxad sensitivity values ranged from 0.29 to 1.93 μg/ml (Figure 1a). There was no strong association between EC_50_ values and exposure to UV or the initial fluxapyroxad concentration. Population L−1 had EC_50_ values from 0.18 to 0.43 μg/ml (Figure 1a).

Most of the fluxapyroxad‐resistant mutants were also less sensitive to fluopyram than isolate IPO323, which has an average EC_50_ value of 0.11 μg/ml (Figure 1b; Table S4). There were significant (*p *<* *.001) differences between populations, with a clear decrease in sensitivity in seven populations (Figure 1b; L−1, L+1, L+2, I+1, I+2, H+1, and H+2), but no change in two non‐UV‐exposed populations (Figure 1b; I‐1 and H‐1). Population I−1 or H−1 had the lowest fluopyram EC_50_ values, ranging from 0.03 to 0.20 μg/ml (Figure 1b). The other populations ranged from 0.28 to >11.0 μg/ml (Figure 1b). Differences in fluopyram sensitivities between IPO323‐derived mutant populations indicate that diverse target‐site mutations may underlie fluxapyroxad resistance in the different populations (Scalliet et al., 2012).

The IPO323‐derived mutants were also less sensitive to carboxin, with EC_50_ values ranging from 4.73 to 146.5 μg/ml (Figure 1c) compared to 1.36 μg/ml for the parental isolate, regardless of the treatment given to the population from which they came (Figure 1c; Table S4). This lower sensitivity to carboxin was expected, as fluxapyroxad and carboxin are SDHI carboxamide fungicides (FRAC, 2015). However, there were significant differences between populations (Figure 1c; *p *<* *0.001).

### 3.3 Detection of SdhB, C, and D variants

**Figure 2:**
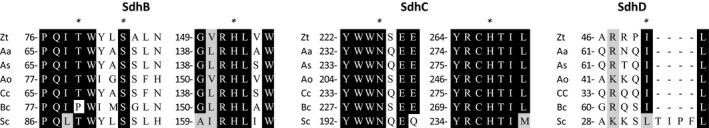
Location of key amino acid substitutions in the *sdh* subunits in IPO323‐derived fluxapyroxad‐resistant mutants. Partial Sdh subunit sequences of *Zymoseptoria tritici* (Zt), *Alternaria alternata* (Aa), *Alternaria solani* (As), *Aspergillus oryzae* (Ao), *Corynespora casiicola* (Cc), *Botrytis cinerea* (Bc), and *Saccharomyces* *cerevisiae* (Sc). Stars indicate positions where amino acid substitutions were found. Conserved residues are shaded in black or grey corresponding to 100% or 80% conservation, respectively

Based on the results of the fluxapyroxad sensitivity test, 57 fluxapyroxad‐resistant mutants were selected and the genes encoding SdhB, C, and D sequenced (Table 1). Mutations were detected in 51 of these, encoding seven different amino acid substitutions. Three amino acid substitutions were detected in the SdhB, exchange from histidine to tyrosine or leucine at codon 267 (B‐H267Y/L) or from asparagine to threonine at codon 225 (B‐N225T). Three other amino acid substitutions were detected in the SdhC, exchange from threonine to isoleucine at codon 79 (C‐T79I), from serine to glycine at codon 83 (C‐S83G), or from histidine to arginine at codon 152 (C‐H152R). One amino acid substitution was detected in SdhD, an exchange from isoleucine to leucine at codon 50 (D‐I50L) (Figure 2). This mutation (D‐I50L) was always found in combination with B‐N225T in four fluxapyroxad‐resistant mutants. Six fluxapyroxad‐resistant mutants, all from the L−1 population, did not carry any amino acid substitutions in the three Sdh subunits forming part of the SDHI binding pocket. Pyrosequencing assays of the L−1 population confirmed the absence of the target‐site mutations found in the other populations. The distribution of substitutions had no obvious association with initial dose or UV exposure (Fig. S2). Most of the amino acid substitutions conferred positive cross‐resistance to fluopyram and carboxin, except amino acid substitution SdhB‐H267Y, which was more sensitive to fluopyram (Table 1; Figs. S2E and H). Fluxapyroxad‐insensitive mutants without changes in the SDHI binding pocket also showed lower sensitivity to both fluopyram and carboxin (Table 1; Figs. S2 and C).

**Table 1:** Succinate dehydrogenase inhibitor (SDHI) sensitivity profiles of IPO323‐derived fluxapyroxad‐resistant mutants carrying different mutations in the SdhB, C, or D encoding genes

Strain/Sdh variant	Codon sequence change	*N*	Fluxapyroxad EC_50_ ± *SE* a	*SD*	RF^b^	Fluopyram EC_50_ ± *SE*	*SD*	RF	Carboxin EC_50_ ± *SE*	*SD*	RF
IPO323	None	1	0.02 ± 0.004	—	—	0.11 ± 0.004	—	—	1.36 ± 0.02	—	—
Nonmutation	None	6	0.29 ± 0.05	0.12	12	0.69 ± 0.15	0.38	6	6.77 ± 0.56	1.38	5
B‐H267L	CAC >CTC/CTT	11	1.57 ± 0.14	0.45	64	4.29 ± 0.34	1.13	40	89.19 ± 6.84	22.68	65
B‐H267Y	CAC > TAC	12	0.76 ± 0.07	0.26	31	0.09 ± 0.01	0.05	0.8	54.23 ± 3.68	12.76	40
B‐N225T, D‐I50L	AAC > ACC, ATT > CTT	4	1.31 ± 0.09	0.22	54	0.97 ± 0.07	0.16	9	7.02 ± 0.47	1.15	5
C‐T79I	ACC > ATC	3	0.90 ± 0.02	0.04	37	0.90 ± 0.12	0.21	8	16.82 ± 1.74	3.02	12
C‐S83G	TCG > GGG	6	1.58 ± 0.09	0.22	65	>11.10	0.00	>104	15.27 ± 1.70	4.18	11
C‐H152R	CAT > CGT	15	1.71 ± 0.16	0.63	70	3.94 ± 0.50	1.94	37	28.74 ± 1.85	7.15	21

a EC_50_ values (μg/ml) are based on the mean of two independent experiments.

b Resistance factors (RFs) of laboratory mutants carrying the same Sdh variant were calculated as the fold change in mean EC_50_ compared with the mean EC_50_ value of the reference *Zymoseptoria** **tritici* isolate IPO323.

### 3.4 Dynamics of SDHI resistant alleles in IPO323‐derived fluxapyroxad‐adapted populations

**Figure 3:**
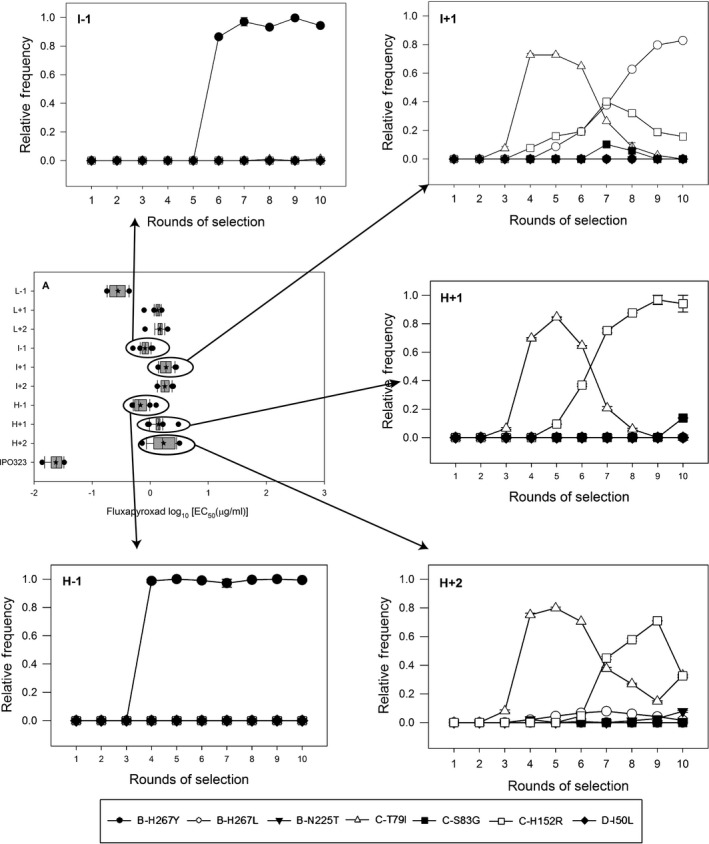
Frequencies of key *SdhB*,* C,* and *D* target‐site mutations in IPO323‐derived fluxapyroxad‐resistant populations during ten rounds of selection (RS) in vitro on YPD amended with increasing concentrations of fluxapyroxad. Fluxapyroxad sensitivity values of IPO323‐derived mutant populations (A). Populations I−1 and I + 1 were exposed to 0.06 μg/ml as starting fungicide concentration. Populations H−1, H + 1, and H + 2 were exposed to 0.08 μg/ml as starting fungicide concentration. Fungicide concentration was increased twofold at every round of selection. Additionally, populations I + 1, H + 1, and H + 2 were exposed to 300 J/m^2^ of UV light at each cycle. Relative allele frequency of positive controls: B‐H267L=1, B‐H267Y=1, B‐N225T=1; C‐T79I=1, C‐S83G=1, C‐H152R=1, and D‐I50L=1. Frequency values are the mean of two technical replicates between replicate pyrosequencing reactions

A continuous replacement of mutants carrying different amino acid substitutions was measured in UV‐exposed populations during subculturing of successive generations on agar with increasing fluxapyroxad concentrations. The mutation underlying B‐H267L was detected in populations I+1 and H+2 at round of selection (RS) 5 or 4, respectively (Figure 3). After an initial detection of 8% at RS5, the frequency of B‐H267L in population I+1 increased gradually to approximately 80% at RS10; whereas in population H+2, B‐H267L increased up to approximately 8% at RS‐7 then decreased to <3% at RS10 (Figure 3). Mutations underlying SdhB‐N225T and SdhD‐I50L simultaneously were only detected in population H+2 (Figure 3). B‐N225T was detected between RS4 and RS9 at low frequencies (2%–5%), and at RS10, a frequency of 7% was measured. D‐I50L was detected at RS3 and RS6 and at RS10 with an approximate allele frequency of 14%. SdhC‐T79I was detected in populations I+1, H+1, and H+2 at RS3 with an approximate allele frequency of 7%, 6%, or 8%, respectively, with an increase in frequency (approximately to 72%, 84%, or 79%, respectively) at RS5. The frequency then decreased to <10% by RS8 and was undetectable after RS9 in populations I+1 and H+1 (Figure 3). A similar pattern of increase and later decrease in allele frequency of C‐T79I (to approximately 15% at RS‐9) was observed in population H+2 (Figure 3). SdhC‐H152R was detected in populations I+1, H+1, and H+2 at RS4 (7%), RS5 (9%) or RS‐6 (˂3%), respectively (Figure 3). The C‐H152R allele frequency increased to approximately 95% in population H + 1 after RS9, whereas in population H + 2, the allele frequency increased to 71% at RS9 then decreased to approximately 32% at RS10. Similarly, C‐H152R increased to approximately 40% at RS7 then decreased to <16% at RS10 in population I+1 (Figure 3). SdhC‐S83G was only detected in population I+1 at RS10 with an approximate allele frequency of 14% (Figure 3).

**Figure 4:**
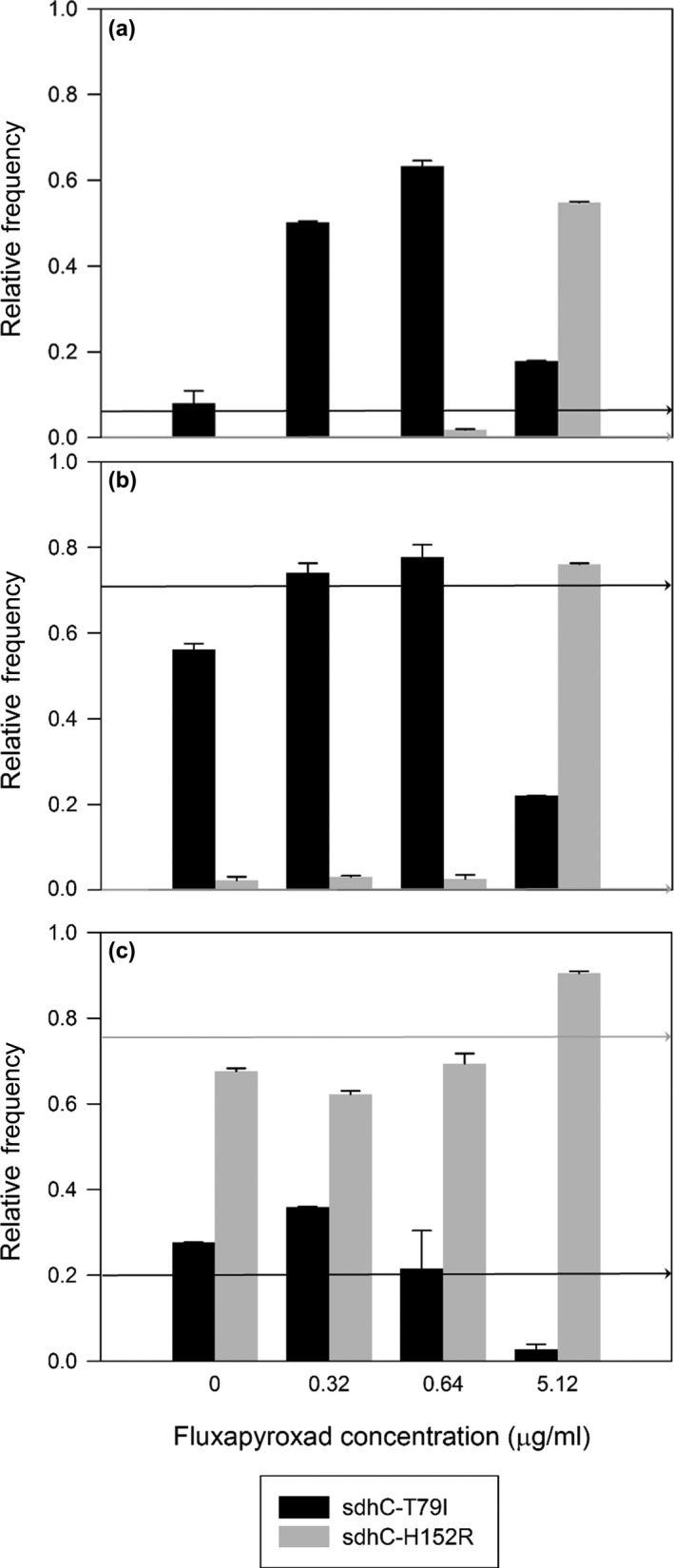
Frequency of SdhC‐T79I and C‐H152R alleles in H + 1 populations grown for seven days at various fluxapyroxad concentrations. Samples were taken from the population after three (a), four (b), and seven (c) rounds of selection (RS). Cultures grew on YPD plates with and without addition of fluxapyroxad for seven days at 21°C in the dark. The arrows indicate the frequency in the parent population from which the samples were taken. Frequency values are the mean of two technical replicates between replicate pyrosequencing reactions. The detection threshold of both SNP detection pyrosequencing assay was 3%. Error bars denote two SEM

In populations not exposed to UV light, no or only a single mutation was detected. The mutation resulting in SdhB‐H267Y was only detected in populations I−1 and H−1 (Figure 4) from RS6 and RS4 onwards, at frequencies ˃80%. No detectable levels of B‐N225T, C‐H267Y/L, C‐T79I, C‐S83G, C‐H152, or D‐I50L alleles were measured in population L−1, confirming the *Sdh* sequencing results of the selected strains (Figs. S2A,B, and C).

### 3.5 Effect of fluxapyroxad concentration on the selection of SdhC‐T79I and C‐H152R variants in population H+1

Changes in frequency of C‐T79I or C‐H152R depended on fungicide concentration during the experiment. When aliquots of the selected populations were regrown without fungicide amendment as for the original DNA extractions, the frequency of C‐T79I and C‐H152R remained comparable to that in the parent population, with a maximum difference of 13% and 2%, respectively, for RS4. However, following further selection at the intermediate fluxapyroxad concentrations of 0.32 and 0.64 μg/ml, the frequency of C‐T79I increased from the low parental level in RS3 (Figure 4a), but was unchanged from its intermediate level in samples from RS4 and RS7 (Figure 4a and b, respectively). At 5.12 μg/ml fluxapyroxad, there was a substantial decrease in frequency of C‐T79I in samples taken from RS4 and RS7 (Figure 4b and c, respectively), but an increase in the sample taken from RS3 (Figure 4a). Frequency of C‐H152R increased sharply at this concentration in samples from all three RS, confirming that it was already present at RS3, but undetectable, below the SNP detection pyrosequencing threshold.

### 3.6 Gene expression

Quantitative RT‐PCR detected a low but significant (*p *<* *0.05) up‐regulation of genes encoding SdhB, C, or D in response to fluxapyroxad treatment in the reference isolate IPO323 (Fig. S3A, Table S5). In the mutant L‐1.7, only genes encoding SdhB or D were significantly (*p *<* *0.001) slightly up‐regulated (between 1.1‐ and 2.1‐fold change; Fig. S3 and Table S6). Reference isolate IPO323 and the L−1.7 showed similar *AOX* gene expression patterns. Overexpression of *AOX* was only significant different in IPO323 when exposed to fluxapyroxad at its approximate EC_50_ and EC_80_ concentrations (Fig. S3, Table S5).

**Figure 5:**
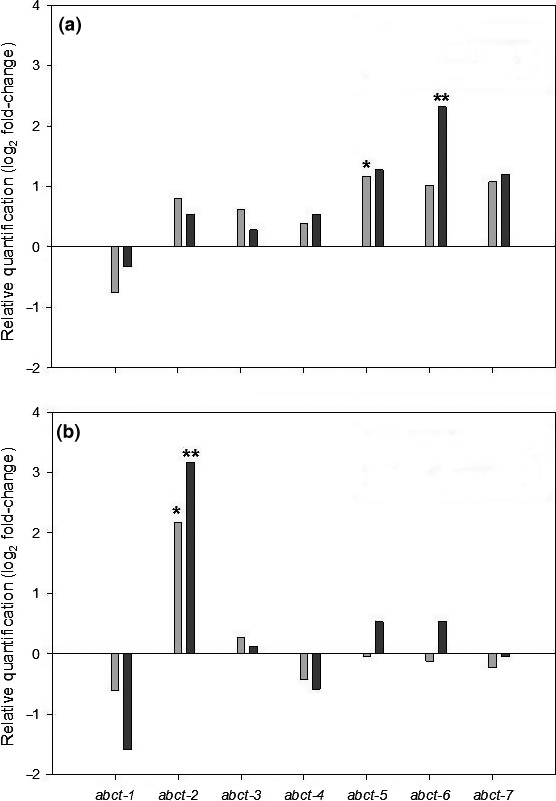
Expression levels of genes encoding putative ATP‐binding cassette transporters (*ABCt*) in the reference *Zymoseptoria** **tritici* isolate IPO323 (a) and the laboratory mutant L−1.7 (b) after 24‐h exposure to their respective fluxapyroxad EC_50_ (light grey) or EC_80_ concentration (dark grey), measured relative to the untreated control using quantitative RT‐PCR. Mean of three biological replicates. Statistical significance for each gene is marked by asterisks (*p <* *.05 or **p <* *.01). See Table S5 and S6 for the means of log_2_ fold‐change data for the treatments with standard error of the difference for the comparisons made

The gene encoding ABCt‐2, a putative ATP‐binding cassette transporter (Goodwin et al., 2011), was constitutively overexpressed in L−1.7 at either the EC_50_ (4.6‐fold change, *p *<* *0.05) or the EC_80_ (9.5‐fold change, *p *<* *0.01) fungicide concentration (Figure 5b, Table S6). Genes *ABCt*‐5 (2.2‐fold change, *p *<* *0.05) and *ABCt*‐6 (5.2‐fold change, *p *<* *0.01) were only up‐regulated in IPO323 in the presence of fluxapyroxad at its EC_50_ or EC_80_ concentration, respectively (Figure 5a, Table S5).

Similarly, a glutathione S‐transferase encoding gene (*GST*‐4) was slightly but significantly (*p *<* *0.05) up‐regulated in L‐1.7 exposed to fluxapyroxad at its approximate EC_50_ (1.3‐fold change, *p *<* *0.05) or EC_80_ (1.9‐fold change, *p *<* *0.01; Fig. S4B, Table S6) concentration. *GST*‐4 was also significantly up‐regulated (4.0‐fold change, *p *<* *0.05 or 10.5‐fold change, *p *<* *0.01), along with *GST*‐1 (2.5‐fold change, *p *<* *0.01 at EC_80_), in the reference isolate IPO323 in the presence of fluxapyroxad (Fig. S4A, Table S5).

Of the major facilitator superfamily efflux drug transporters, *MSF*‐2 (5.3‐fold change, *p *<* *0.05 or 12.9‐fold change, *p *<* *0.01) and *MSF*‐6 (2.8‐fold change, *p *<* *0.05 or 8.8‐fold change, *p *<* *0.01) were significantly up‐ in the ;regulatedreference isolate IPO323 (Fig. S5A, Table S5). In the fluxapyroxad‐resistant mutant L−1.7, no *MSF* encoding genes were significantly (*p *<* *0.05) up‐ or down‐regulated (Fig. S5B,Table S6).

## 4 DISCUSSION

As the SDHI sensitive isolate *Z. tritici* IPO323 was used as progenitor of all populations, no external genotypes entered the mutant populations and no genetic exchange among populations occurred, so mutation was the only source of genetic variability. Therefore, selectively advantageous mutations conferred steadily reducing sensitivity to fluxapyroxad over time in all nine surviving populations of *Z*. *tritici*. Seven of the nine populations were also less sensitive to fluopyram or carboxin; two were more sensitive to fluopyram.

### 4.1 Resistance mechanisms in IPO323‐derived fluxapyroxad‐resistant mutants

Distinct molecular mechanisms conferred fluxapyroxad resistance with positive or negative cross‐resistance to fluopyram. Mutations leading to amino acid substitutions in the SDHI binding pocket of the target protein (SDH) were the most common. Mutations in the *SdhB* gene resulted in amino acid changes at codon 225 (N225T) or 267 (H267Y/L). The variant B‐H267L conferred resistance to fluxapyroxad, carboxin, and fluopyram, whereas B‐H267Y increased sensitivity to fluopyram. This negative cross‐resistance of B‐H267Y was reported before for *Z. tritici* by Fraaije et al. (2012) and Scalliet et al. (2012) and has also been reported for other plant pathogens (see Sierotzki & Scalliet, 2013). In one mutant, two mutations leading to B‐N225T and D‐I50L were found simultaneously. This haplotype was associated with a high level of insensitivity to fluxapyroxad and lower levels of insensitivity to carboxin and fluopyram (Table 1). Scalliet et al. (2012) reported B‐N225H/I in laboratory mutants, conferring lower sensitivity to carboxin, isopyrazam, fluopyram, and boscalid. An equivalent alteration in *Botrytis cinerea*, B‐N230I, has also been linked with SDHI resistance (Leroux, Gredt, Leroch, & Walker, 2010). Equivalent mutations to D‐I50L are not known and the precise role of D‐I50L, alone or in combination with B‐N225T, in SDHI binding needs further studies. Mutants carrying C‐T79I, C‐S83G, or C‐H152R are insensitive to fluxapyroxad, fluopyram, and carboxin (Table 1). Scalliet et al. (2012) reported that C‐T79I, C‐H152R, or C‐S83G variants can confer resistance to a range of SDHIs (i.e., carboxin, isopyrazam, fluopyram, and boscalid). Interestingly, they reported that C‐S83G variants have higher levels of resistance to fluopyram than variants C‐T79I or C‐H152R. Similar results were found in our study.

Fluxapyroxad‐insensitive isolates with no target‐site (SdhB, C, or D) alterations were also less sensitive to fluopyram and carboxin. The gene expression study indicated that overexpression of an ABC transporter may contribute to the phenotype. The *ABCt*‐2 gene, encoding a putative *Z. tritici* ABC transporter (Goodwin et al., 2011), was constitutively overexpressed up to 9.5‐fold in the fluxapyroxad‐resistant IPO323 mutant L−1.7 (Figure 5b). Similarly, Cowen et al. (2002) reported overexpression of an ABC transporter in a mutant population of *C. albicans* grown for 330 generations in presence of fluconazole, without mutations in the target protein. Overexpression of ABC transporters was also detected in *S. cerevisiae* after 400 generations at increased concentrations of fluconazole (Anderson et al., 2003). The overexpression of the *ABCt‐2* gene indicates the ability of *Z. tritici* to develop resistance mechanisms to single‐site fungicides through nontarget‐site mutations. A similar mechanism has also already been reported in field isolates, with overexpression of *MgMfs1*, encoding an MFS transporter, conferring insensitivity to different fungicides, including SDHIs and azoles (Omrane et al., 2015).

### 4.2 Emergence and evolution of in vitro resistance to fluxapyroxad

Emergence of amino acid substitutions in the Sdh target protein occurred in parallel at similar rates in the UV light exposed populations I+1, H+1, and H+2 (Figure 3). Early in the experiment, the amino acid substitution C‐T79I emerged in these populations after adaptation to three RS at increasing rates of fluxapyroxad. However, C‐T79I started to be outcompeted by C‐H152R after five or six selection rounds in populations H+1 or H+2, respectively. Similarly, C‐T79I was superseded by B‐H267L after five RS in population I+1. Subsequently, mutants carrying C‐S83G or a double mutant (B‐N225T and D‐I50L) started to increase in frequency at the end of the experiment in populations H+1 and H+2.

The sequence of mutants carrying distinct amino acid substitutions in the SDHI binding pocket of the Sdh complex, C‐T79I followed by C‐H152R or B‐H267L, indicates haplotype replacement of Sdh variants, each having arisen independently from the parental strain, over the course of the experiment. Clonal or lineage replacement takes place in asexual populations where mutations conferring beneficial advantages arise independently and compete with others leading to a succession of genotypes, each one fitter than its immediate predecessor, until the best adapted remains under specific conditions or threats (Muller, 1932). Atwood, Schneider, and Ryan (1951) reported a periodic replacement of strains (i.e., lineage replacement) in *E. coli* populations evolving in vitro for 1,000 generations under histidine‐limited conditions. They observed a repeated cycle of clonal strain substitutions between histidine‐required (h−) and histidine‐independent (h+) strains in mixed *E. coli* populations consisting of various ratios of h+/h−, inferring that clonal replacement took place approximately 4.5 times during the experiment. Recently, Albrecht, Jatzwauk, Slickers, Ehricht, and Monecke (2011) reported replacement of methicillin‐resistant *Staphylococcus aureus* (MRSA) strains in human patients during a period of 11 years in German hospitals. They detected four distinct MRSA strains with their frequencies fluctuating during the study. For example, a strain named CC22‐MRSA‐IV was detected in 2001 and increased in frequency up to approximately 58% in 2010; other strains such CC45‐MRSA‐IV decreased approximately 58% frequency between 2002 and 2010.

Lineage replacement may be due to directly mutation‐limited evolution, in which the fittest available mutation is selected but chance mutations later generate a fitter genotype; it may be due to compensatory mutations, in which a mutation may have arisen early in the experiment but carries fitness penalties until compensatory mutations emerge; or it may be due to changing selection over time, such as the increasing fungicide concentrations in the present experiment. By re‐selecting the populations from different RS with different fungicide doses, we show that in the experimental evolution of resistance to SDHI fungicides in *Z. tritici*, lineage replacement was driven by changes in fungicide concentration. When the population from an early round of selection (RS3) was exposed to a much higher fungicide concentration (5.12 μg of fluxapyroxad/ml), variant C‐H152R was selected (Figure 4). This shows that C‐H152R was already present after three RS, but only reaches detectable frequencies under selection at a high fungicide concentration (Figures 3 and 4). The C‐H152R mutants present in RS3 were immediately selected when transferred to higher fungicide concentrations, indicating that no further, compensatory mutations were needed. This suggests that mutants carrying C‐H152R are unable to compete and less fit than those carrying C‐T79I at fungicide concentrations <5.12 μg/ml. Thus, mutations might emerge simultaneously but a given mutation will only increase in frequency and dominate the population under particular fungicide concentrations (Van den Bosch, Paveley, Shaw, Hobbelen, & Oliver, 2011). In a mixed population of wild‐type, C‐T79I and C‐H152R variants, the optimal mutant‐selective window for C‐T79I is between 0.32 and 0.64 μg/ml, whereas the mutant‐selective window for C‐H152R starts between 0.64 and 5.12 μg/ml, under the growth conditions used here. Further competition experiments could define the mutant‐selective windows more precisely both in vitro and in planta.

Selection by increasing fungicide doses can lead to sequential selection in a way that constant doses would not. This is because some strains less sensitive or resistant to SDHI fungicides appear to be less fit due to reduced SDH enzyme activity. For example, C‐H152R was associated with lower SDH enzyme activity (47% of covalent FAD, 48% activity of MTT/PMS, and 22% residual activity of Qo/DCPIP) than the C‐T79I variant (73% of covalent FAD, 106% activity of MTT/PMS, and 26% residual activity of Qo/DCPIP) (Scalliet et al., 2012). Therefore, if lower doses are initially used, strains with lower levels of resistance (e.g., C‐T79I) may initially be selected, after which fungicide dose could be increased to regain some degree of control, until other more‐resistant strains (e.g., C‐H152R, C‐S83G, or B‐H267L) are selected at higher doses. The patterns of genotype replacement in our data are consistent with the hypothesis that some alleles are only selectively advantageous in restricted fungicide concentration ranges.

If not mitigated by changes elsewhere in the genome, this fitness penalty could play a key role in the development of resistance management strategies. Although higher doses of SDHIs can reduce SLB disease symptoms slightly more effectively (Dooley, Shaw, Spink, & Kildea, 2016), using lower doses of SDHIs may reduce selection for strains with higher resistance levels.

In contrast, in two populations, I−1 and H−1, both exposed to initially greater concentrations of fluxapyroxad (0.06 or 0.08 μg/ml) but not to UV light, only a single mutation resulting in amino acid substitution SdhB‐H267Y arose after four or five RS and reached a high frequency, close to fixation (Figure 3). Additionally, in population L−1, not exposed to UV, fluxapyroxad resistance was associated with overexpression of *abc*t *2* and no further mutations were detected over the course of the experiment. This suggests that lineage replacement is less common in a mutation‐limited system and/or takes much longer to develop.

### 4.3 Comparison of SDHI resistance development in the field and in vitro

Adaptation to fungicides by target‐site or nontarget‐site alterations has already occurred in field populations of *Z. tritici* (see Clark, 2006; Cools & Fraaije, 2013; Fraaije et al., 2005; Omrane et al., 2015; Torriani et al., 2009). With regard to SDHI resistance, strains carrying amino acid substitutions SdhB‐N225T, B‐T268I, C‐T79N, C‐W80S, and C‐N86S, conferring low levels of insensitivity, have been reported in field populations (FRAC, 2015, 2016). In our evolutionary study, resistance to fluxapyroxad emerged first by substitution of tyrosine by isoleucine at codon 79 of SdhC, which is the same position where T79N was found in field isolates. The reported C‐T79N field strains have low resistance factors to different SDHIs. In our study, in vitro sensitivity assays indicated that the C‐T79I variants had resistance factors of 37, 8, and 12 to fluxapyroxad, fluopyram, and carboxin, respectively (Table 1). Interestingly, one field strain carrying C‐T79I was found in Hampshire (UK) during late summer in 2016 and is likely to spread further in the absence of fitness costs (B. A. Fraaije, unpublished). Interestingly, a single field strain carrying B‐N225T has been reported (FRAC, 2015). Mutants carrying B‐N225T and D‐I50L showed higher resistance factors for fluxapyroxad than C‐T79I mutants, but the resistance factors for fluopyram and carboxin were still low. Our studies and Scalliet et al. (2012) showed that several amino acid substitutions (e.g., B‐H267L, C‐S83G, and C‐H152R) with high resistance factors to all SDHIs might emerge in *Z. tritici* field populations. Indeed, C‐H152R strains conferring high levels of resistance (RF >100) to bixafen, penthiopyrad, and fluxapyroxad were recently found in Z. *trititici* field populations sampled in Ireland (Dooley, Shaw, Mehenni‐Ciz et al., 2016) and the UK (B. A. Fraaije, unpublished). Our study indicates that a fitness penalty (inability to compete with other strains at lower fungicide concentrations) might be associated with this mutation which in the absence of further adaptation through other genetic chances might delay the further spread of this Sdh variant in field populations.
