# Supplementary material for: Dose‐dependent selection drives lineage replacement during the experimental evolution of SDHI fungicide resistance in Zymoseptoria tritici
Source: Evol Appl. 2017 Sep 3;10(10):1055–66. doi: 10.1111/eva.12511 (PMC5680630; doi:10.1111/eva.12511)
Supplement: Supplementary file 1 [file EVA-10-1055-s001.docx]

**Supplementary information**

**Material and Methods**

**Fungicide sensitivity testing**

Spore suspensions of mutants, grown on YPD plates for seven days at 15 °C in the dark, were harvested and adjusted at a concentration of 2.5×10^4^ spore ml^-1^ in SDW after counting spore suspensions under a microscope. Aliquots of 100 µL spore suspension were added to 100 µL double strength sabouraud dextrose broth (SDB; Oxoid Basingstoke, UK) amended with increasing fungicide concentrations in clear, flat-bottomed, 96 well cell culture plates (Greiner Bio-One, Frickenhausen, Germany). Increasing concentrations of fluxapyroxad, fluopyram (0.002, 0.005, 0.015, 0.046, 0.14, 0.4, 1.2, 3.7, 11.1, 33.3 and 100 μg ml^-1^) or carboxin (0.06, 0.05, 0.14, 0.41, 1.23, 3.70, 11.11, 33.33, 100, 300 and 900 µg ml^-1^) were used. Technical grade fluopyram (10 mg ml^-1^; 32462 Fluka, Sigma-Aldrich, UK) or carboxin (50 mg ml^-1^; 45371 Fluka, Sigma-Aldrich, UK) was dissolved in dimethylsulphoxide (DMSO) before dilution in the liquid media. Formulated fluxapyroxad as emulsifiable concentrate (62.5 g l^-1^ EC; BASF, Ludwigshafen, Germany) was dissolved in sterile distilled water at 10 mg ml^-1^ as a stock concentration before dilution.

**SNP detection pyrosequencing assays**

Primers (see supplementary Table S2) targeting DNA regions harbouring *SdhB*, *C* or *D* mutations linked to SDHI resistance were designed with the Pyrosequencing Assay Design software (version 1.0.6; Biotage, Uppsala, Sweden) using the following sequences to analyze: ggaa/cctcggaggagtacctcggaccagct (B-N225T), gcca/tcaccattctgaattgctcaaggacc (B-H267L), gcc/tacaccattctgaattgctcaaggac (B-H267Y), a/gttatttgcggtttgtagattgcgag (C-T79I), a/cgaggtaccaggttatttgcggtttg (C-S83G), c/tgcctcactccattcaacgaatgaaa (C-H152R) and g/taggtctccgcgcagtggtctggaag (D-I50L). Amplification of these target regions was carried out on a Biometra T3000 thermocycler (Göttingen, Germany) in a final volume of 50 µL PCR reaction containing 50 ng of template DNA, 0.2 units of One*Taq* DNA polymerase (New England BioLabs), 1x One*Taq* standard reaction buffer with 200 µM dNTP and 0.2 µM for each primer (Table 2). Thermocycling conditions were 94 °C initial denaturation for 30 seconds, 40 cycles at 94 °C for 15 seconds, 56 °C for 15 seconds and 72 C for 15 seconds, and a final DNA extension at 68 °C for 5 minutes. Biotin labelled PCR products were captured with Streptavidin Sepharose HP beads (GE Healthcare, Uppsala, Sweden), and made single-stranded according to the protocol of [Carter et al. (2013)](#_ENREF_1). Beads with single-stranded PCR products were transferred into a Pyromark Q96 HS plate (Qiagen) containing 0.15 µL 100 µM of sequencing primer (Supplementary Table S2) and 44.85 µL of Pyromark annealing buffer (Qiagen). Pyrosequencing reactions were carried out on a PyroMark Q96ID (Biotage, Uppsala, Sweden) using a PyroMark Gold Q96 Reagent kit (Qiagen) according to the manufacturer’s instructions using the following dispensation orders: cgacgtcga (B-N225T), tgcatgcac (B-H267L), tgctgacac (B-H267Y), cagctatgc (C-T79I), tactgagta (C-S83G), actagctca (C-H152R) and cgtcagtct (D-I50L).

**Gene expression studies**

Spores of IPO323 and mutant L-1.7 were grown on YPD plates at 15 °C in the dark for seven days. Flasks containing 100 mL of SDB amended with formulated fluxapyroxad (62.5 g l^-1^ EC, BAS 700 00 F, BASF) to give a final concentration of 0.04 or 0.19 and 0.39 or 1.56 µg ml^-1^ were inoculated with spores (5×10^5^ spores ml^-1^) of IPO323 or L-1.7, respectively. Formulated fluxapyroxad (0.1 mg ml^-1^) was dissolved in sterile distilled water before adding to the culture medium. IPO323 or L-1.7 grown for 24 hours in the absence of fungicide was used as the untreated control. After 24 hours’ growth at 21 °C in the dark at 200 rpm, fungal biomass was harvested by vacuum filtration and snap frozen in liquid nitrogen.

‘Total’ RNA from triplicate untreated and treated samples was extracted from freeze-dried tissue with TRIzol reagent (Invitrogen, Carlsbad, California, USA) following the manufacturer’s protocol, with 1-bromo-3-chloropropane (Molecular Research Center, Cincinnati, USA) as the phase separating agent. RNA was purified by precipitating overnight in 4 M lithium chloride (Sigma-Aldrich, Steinheim, Germany) at -20 °C. Five micrograms of total purified RNA was reverse transcribed with random primers using the High-Capacity cDNA Reverse Transcription kit (Applied Biosystems, Foster City, California, USA) according to the supplier’s instructions and diluted one in ten.

Quantitative real-time PCR reactions were carried out using SYBR® Green JumpStart Taq ReadyMix (Sigma-Aldrich, Missouri, USA) in a final volume of 20 µL containing 5 µL of diluted cDNA and 0.25 µM of each primer (Supplementary Table S3). Thermal cycling conditions were 95 °C for 2 min, 40 cycles at 95 °C for 15 seconds, 58 °C for 30 seconds and 72 °C for 40 seconds. Fluorescence was measured at each 72 °C elongation step. Reactions were carried out on a Stratagene Mx3000P QPCR System (Agilent Technologies, USA).

TABLE S1 Primers used to amplify and sequence the succinate dehydrogenase (Sdh) subunit B, C or D encoding genes in *Z. tritici* IPO323-derived mutants.

| **Primer ID/Source** | **Target gene** | **Sequence (5’ – 3’)** | **Application** |
| --- | --- | --- | --- |
| SDHB 752F^1^ | *sdhB* | TAAACACTCCACGCCTCACG | PCR amplification |
| SDH2_ST1R^2^ |  | GTCTTCCGTCGATTTCGAGAC | PCR amplification |
| Mgsdhbf1^3^ |  | ACTCTTCTCACATACCACACA | Sequencing |
| Mgsdhbr1^3^ |  | CTTTCCAATCATCTCGTTCCAT | Sequencing |
| SDHC 888F^1^ | *sdhC* | TCCTGTCCTGTGATCCTGGA | PCR amplification |
| SDHC 1768R^1^ |  | TCCCTTGGGTCCTGATGTAC | PCR amplification |
| Mgsdhcf1^3^ |  | GGCACATCGCGTCTCACG | Sequencing |
| SDHD_NEW1RMG^2^ | *sdhD* | GGCATCATCGTCAAGCAAG | PCR amplification |
| SDHD 1826R^1^ |  | CAATTCTTCTTGGCAGCAACA | PCR amplification |
| Mgsdhdf1^3^ |  | CTCACCCTCACCGTCGCC | Sequencing |

^1^Hawkins, N.J. (2013) (unpublished)

^2^[Dubos et al. (2013)](#_ENREF_2)

^3^[Fraaije et al. (2012)](#_ENREF_3)

TABLE S2 Primers used in SNP detection pyrosequencing assays to determine the frequency of key SDHI target-site mutations in the *Zymoseptoria tritici* *SdhB*, *C* and *D* genes.

| **Primer ID** | **Amino acid alteration** | **Sequence (5' - 3')** | **Application** |
| --- | --- | --- | --- |
| 225S | SdhB - N225T | GCCCATCCTACTGGT | Sequencing primer |
| 225F |  | ATCTTGCCCATCCTACTGGTG | PCR amplification |
| 225BioR |  | CCATCGGTATGACTGGAGAAG | PCR amplification |
| 267S | SdhB - H267Y/L | CATGAGCTTGTACCGAT | Sequencing primer |
| 267BioR^1^ |  | CAGGTCCTTGAGCAATTCAGA | PCR amplification |
| 267F |  | CGCACTCAACAACAGCATGA | PCR amplification |
| 79S | SdhC - T79I | GCCGAGAGGTACCAG | Sequencing primer |
| 79BioF |  | GCCCCACCTCGCAATCTA | PCR amplification |
| 79R |  | TGAGGGCCGAGAGGTACCA | PCR amplification |
| 83S | SdhC - S83G | CGCGGTTGAGGGCCS | Sequencing primer |
| 83BioF |  | CCCCACCTCGCAATCTACAAAC | PCR amplification |
| 83R |  | CCGTGACGCGGTTGAGGG | PCR amplification |
| 152S | SdhC - H152R | CGTATCCCACACCAAA | Sequencing primer |
| 152BioF |  | CCGGTGACGTTTCATTCGTT | PCR amplification |
| 152R |  | TAATCATACTCGCCGTATCCCACA | PCR amplification |
| 50S | SdhD - I50L | GGAAGAGGAGGGAGAA | Sequencing primer |
| 50BioF |  | TCCGGCTTCCAGACCACTG | PCR amplification |
| 50R |  | TTGCGGAAGAGGAGGGAGA | PCR amplification |

^1^Bio means 5’ biotin labelled primer.

TABLE S3 Primer sets (5’-3’) used to determine changes in mRNA levels after fluxapyroxad exposure using qualitative RT-PCR

| Gene ID | JGI gene ID^1^ | Forward | Reverse |
| --- | --- | --- | --- |
| *β-tubulin* | e_gw1.1.861.1 | CGCATGATGGCCACCTTCTC | GCAGAAGGTCTCGTCGGAATT |
| *sdhB* | estExt_Genewise1Plus.C_chr_70866 | CGAAGACAAAGACCTTCCACATCT | TCAGAGCATCCAACATCATAGGA |
| *sdhC* | fgenesh1_kg.C_chr_8000081 | CTTCGGACTCCTCTACCTCG | CACACCAAATGCCTCACTCC |
| *sdhD* | fgenesh1_kg.C_chr_4000004 | TCCGCCATCACCGACTACTT | CCCGCAGTCAAACCAATATCG |
| *aox* | estExt_Genewise1Plus.C_chr_60389 | GCACAAGGCGTCTTCTTCAA | GGCGATTTCACGGGTGTAAGT |
| *abct-1* | e_gw1.9.8.1 | GCTCCCAGATAAGGCCGAAGA | TTGTACACCAGCCCATCCGATA |
| *abct-2* | estExt_Genewise1.C_chr_41071 | GCCATCCCTATCGCTCTCATC | GAAGCCAGACAGTTCCCATCAT |
| *abct-3* | estExt_Genewise1Plus.C_chr_11491 | GGTGACAAGGTGGATGATGATC | CAGTCGGAGCAAAGCAGAATC |
| *abct-4* | estExt_Genewise1.C_chr_51261 | AGACTGGGCTATGCGGAAATC | GGTGAGCGTTGGAAAGAAGAG |
| *abct-5* | e_gw1.6.23.1 | GGGACAACAACACAGACTTCAA | CGGTATGCAGTGAAGTGATGAT |
| *abct-6* | estExt_fgenesh1_pm.C_chr_20184 | GACGTGTGCAGTTGTGTATGG | GGGTCTCGGTCTCCTGTTTAAG |
| *abct-7* | estExt_Genewise1.C_chr_80997 | AAGCCGAGAGTGAGATGAAGAG | TAGACGGATCCATGCCATCGATA |
| *mfs-1* | estExt_fgenesh1_kg.C_chr_70313 | GTGGATGGTTATTGGATACCTCAT | CCCACAATGAGAGCGATTTGA |
| *mfs-2* | e_gw1.1.2469.1 | GGACGAATTGGGTTGTGGTGATT | CCTCCTCCTTCCTCTTCCTCTT |
| *mfs-3* | estExt_Genewise1.C_chr_11110 | GCCCATTGAGCGAGGTGTAT | GCGAGGCTATCAAGAGACCTTTG |
| *mfs-4* | e_gw1.7.95.1 | GCCAGACCCATGTACTCTAAGT | CTTCCTTATTCTCGACCCGTAGA |
| *mfs-5* | estExt_Genewise1Plus.C_chr_22238 | GGCGACGTTCACACATACATAC | GAGGTCAGAAATCCGGCTTGAA |
| *mfs-6* | gw1.1.2530.1 | GGAGTTCATGCCGAGGAAGAG | GCCATTCCCAATCGCCACAAT |
| *mfs-7* | estExt_Genewise1Plus.C_chr_12354 | GCAACTCATTCTCTCTGCATTCT | ACTTCCAATCGCCAGTTTCTTTG |
| *gst-1* | estExt_Genewise1Plus.C_chr_32118 | CATCATGAGACCATACCCGAAAG | GTACTTGCTGCCGAAGAGTTT |
| *gst-2* | estExt_fgenesh1_kg.C_chr_30405 | CATCAGCCAGAACATCCAGAAG | ACGGGTCACATAGGACAGAATG |
| *gst-3* | estExt_fgenesh1_pg.C_chr_30174 | CTCGGATCGCATTCTTCCATCAG | ATCTGCACCGTTTCCTTCGATTT |
| *gst-4* | fgenesh1_pm.C_chr_1000191 | GCCGAGAATGAGGGACCTTA | ATCCTTCATGATCCTTCCATACAC |
| *gst-5* | fgenesh1_pg.C_chr_7000293 | TTGTCAATCGAAGCGTCCACTA | CCTCCACCATCTCAGCGAATG |
| *gst-6* | estExt_fgenesh1_kg.C_chr_70091 | CGGGTAATGGAAAGGGACGATAT | ACCCACTTTAACAAATGCGGAAAC |
| *gst-8* | estExt_fgenesh1_kg.C_chr_10601 | GATACATTCACGGACGGCAAAC | GATACATTCACGGACGGCAAAC |

^1^The Join Genome Institute (JGI) fungal program ([Grigoriev et al., 2011](#_ENREF_4))

TABLE S4 Succinate dehydrogenase inhibitor sensitivity profiles of *Z. tritici* IPO323-derived laboratory mutant populations obtained after ten rounds of *in vitro* selection on YPD plates amended with 0.04 (L-1, L+1, L+2), 0.06 (I-1, I+1, I+2) or 0.08 (H-1, H+1, H+2) µg ml^-1^ of fluxapyroxad as starting point with or without 300 J m^-2^ UV light exposure**.**

| Population | **UV**  **exposure** | **N** | **Fluxapyroxad**  **EC_50_ ± SE^1^** | **SD** | **Fluopyram**  **EC_50_ ± SE** | **SD** | **Carboxin**  **EC_50_ ± SE** | **SD** |
| --- | --- | --- | --- | --- | --- | --- | --- | --- |
| L-1 | - | 20 | 0.29 ± 0.02 | 0.09 | 0.61 ± 0.06 | 0.26 | 6.78 ± 0.42 | 1.88 |
| L+1 | + | 20 | 1.35 ± 0.01 | 0.07 | 3.69 ± 0.11 | 0.51 | 75.93 ± 2.90 | 12.97 |
| L+2 | + | 20 | 1.45 ± 0.02 | 0.11 | 2.92 ± 0.35 | 1.56 | 20.80 ± 1.88 | 8.42 |
| I-1 | - | 20 | 0.83 ± 0.03 | 0.14 | 0.06 ± 0.004 | 0.02 | 58.75 ± 1.79 | 8.02 |
| I+1 | + | 20 | 1.93 ± 0.10 | 0.44 | 4.82 ± 0.30 | 1.35 | 76.08 ± 7.14 | 31.95 |
| I+2 | + | 20 | 1.82 ± 0.08 | 0.37 | 10.35 ± 0.86 | 1.86 | 31.81 ± 8.83 | 39.48 |
| H-1 | - | 20 | 0.71 ± 0.05 | 0.21 | 0.09 ± 0.01 | 0.04 | 46.63 ± 1.61 | 7.21 |
| H+1 | + | 20 | 1.44 ± 0.10 | 0.43 | 5.48 ± 0.86 | 3.87 | 20.15 ± 1.13 | 5.06 |
| H+2 | + | 20 | 1.81 ± 0.17 | 0.78 | 2.60 ± 0.52 | 2.33 | 24.29 ± 4.04 | 18.08 |

^1^EC_50_ values (µg ml^-1^) are the mean of two independent experiments.

TABLE S5 ANOVA of log_2_ fold-change in gene expression of various target genes in *Z. tritici* isolate IPO323 after different fluxapyroxad exposures relative to the untreated control, showing standard errors of difference (SED) between means of 5 degrees of freedom.

| **Target gene ID** | **EC_50_** | **EC_80_** | **SED** |
| --- | --- | --- | --- |
| *sdhB* | +1.42 | +1.84 | 0.458 |
| *sdhC* | +1.11 | +1.87 | 0.441 |
| *sdhD* | +1.41 | +1.83 | 0.488 |
| *aox* | -1.73 | +0.84 | 0.537 |
| *abct-1* | -0.76 | -0.33 | 0.461 |
| *abct-2* | +0.80 | +0.54 | 0.595 |
| *abct-3* | +0.61 | +0.28 | 0.503 |
| *abct-4* | +0.39 | +0.54 | 0.517 |
| *abct-5* | +1.17 | +1.28 | 0.477 |
| *abct-6* | +1.02 | +2.32 | 0.448 |
| *abct-7* | +1.08 | +1.21 | 0.564 |
| *gst-1* | -0.66 | +1.30 | 0.436 |
| *gst-2* | -0.24 | +0.30 | 0.477 |
| *gst-3* | +0.11 | +0.39 | 0.638 |
| *gst-4* | +1.92 | +2.89 | 0.616 |
| *gst-5* | +0.82 | +1.61 | 0.643 |
| *gst-6* | -0.44 | +0.51 | 0.426 |
| *gst-8* | +0.13 | -1.21 | 1.456 |
| *mfs-1* | +0.03 | +0.10 | 0.631 |
| *mfs-2* | +2.24 | +3.68 | 0.777 |
| *mfs-3* | +0.35 | +0.34 | 0.671 |
| *mfs-4* | -0.95 | -0.84 | 0.500 |
| *mfs-5* | +0.12 | -0.27 | 0.598 |
| *mfs-6* | +1.46 | +3.07 | 0.610 |
| *mfs-7* | -0.46 | +0.21 | 0.495 |

TABLE S6 ANOVA of log_2_ fold change in gene expression of various target genes in *Z. tritici* laboratory mutant L-1.7 after different fluxapyroxad exposures relative to the untreated control, showing standard errors of difference (SED) between means of 5 degrees of freedom.

| **Target gene ID** | **EC_50_** | **EC_80_** | **SED** |
| --- | --- | --- | --- |
| *sdhB* | +0.23 | +0.84 | 0.175 |
| *sdhC* | +0.45 | +0.31 | 0.502 |
| *sdhD* | +0.37 | +1.05 | 0.195 |
| *aox* | -0.18 | +0.58 | 1.647 |
| *abct-1* | -0.61 | -1.59 | 0.745 |
| *abct-2* | +2.17 | +3.17 | 0.622 |
| *abct-3* | +0.27 | +0.12 | 0.424 |
| *abct-4* | -0.43 | -0.60 | 0.469 |
| *abct-5* | -0.04 | +0.53 | 0.683 |
| *abct-6* | -0.13 | +0.53 | 0.361 |
| *abct-7* | -0.23 | -0.05 | 0.361 |
| *gst-1* | -0.37 | -0.96 | 0.514 |
| *gst-2* | -0.35 | -1.20 | 0.403 |
| *gst-3* | -0.05 | -1.00 | 0.574 |
| *gst-4* | +0.460 | +1.32 | 0.158 |
| *gst-5* | -0.04 | -0.22 | 0.433 |
| *gst-6* | -0.10 | -0.36 | 0.381 |
| *gst-8* | -0.35 | -0.06 | 0.331 |
| *mfs-1* | +0.29 | -0.08 | 0.489 |
| *mfs-2* | +0.12 | +0.43 | 0.989 |
| *mfs-3* | -0.14 | -0.47 | 0.847 |
| *mfs-4* | -0.11 | +0.69 | 0.567 |
| *mfs-5* | -0.11 | -0.09 | 0.642 |
| *mfs-6* | -0.63 | +0.54 | 1.272 |
| *mfs-7* | +0.01 | -0.41 | 1.277 |

FIGURE S1 Progression of *Z. tritici* IPO323-derived populations through growth on YPD plates amended with up to ten successively doubled concentrations of fluxapyroxad starting from 0.04 µg ml^-1^ (A), 0.06 µg ml^-1^ (B) or 0.08 µg ml^-1^ (C). Populations L+1, L+2, I+1, I+2, H+1 and H+2 were also exposed to UV light (300 J m^-2^) at the start of each generation.

**
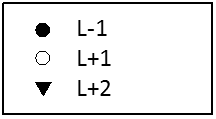
**

**
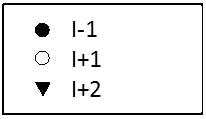
**

**
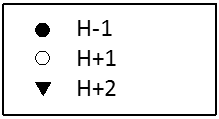
**

FIGURE S2 Detection of SdhB, SdhC and SdhD amino acid alterations associated with SDHI insensitivity in IPO323-derived mutant populations of *Z. tritici* after ten rounds of selection *in vitro* on YPD amended with 0.04 (A, B, C), 0.06 (D, E, F) or 0.08 (G, H, I) µg of fluxapyroxad ml^-1^ as starting point. B-H267Y, B-H267L and B-N225T; C-T79I, C-S83G and C-H152R; D-I50L;
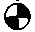
 no *Sdh* mutations. Fungicide concentration was increased two-fold during each round of selection. Populations L+1, L+2, I+1, I+2, H+1 and H+2 were also exposed to UV light (300 J m^-2^) at the start of each generation. SDHI sensitivities of the parent isolate IPO323 are indicated by the arrows.


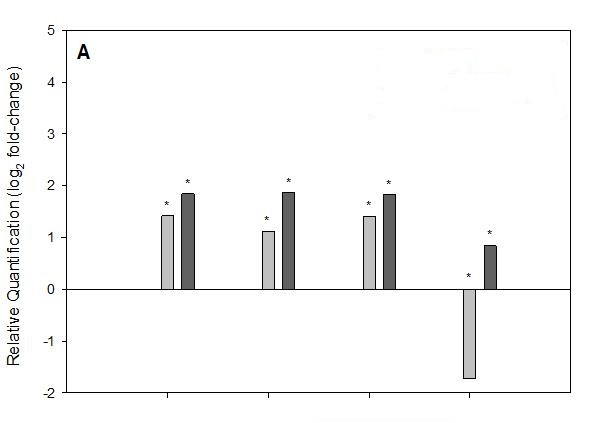

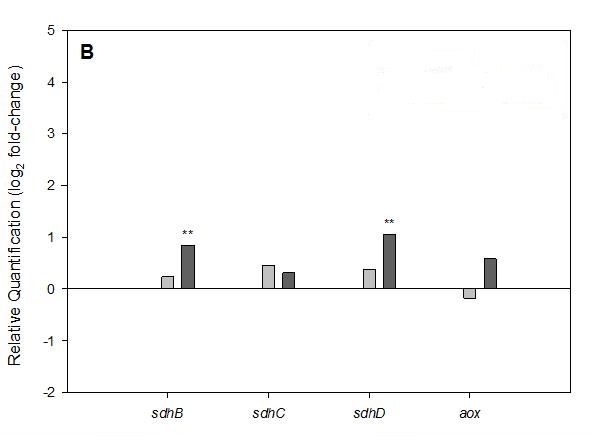


FIGURE S3 Expression levels of genes encoding succinate dehydrogenase (*SdhB*, *C* and *D*) and alternative oxidase (*aox*) in the reference *Z. tritici* isolate IPO323 (A) and the laboratory mutant L-1.7 (B) after 24 h exposure to their respective fluxapyroxad EC_50_ (light grey) or EC_80_ concentration (dark grey), measured relative to the untreated control using quantitative RT-PCR. Mean of three biological replicates. Statistical significance for each gene is marked by asterisks (*=*p*<0.05 or **=*p*<0.01). See Supplementary Table S5 and S6 for the means of log_2_ fold-change expression data for the treatments with standard error of the difference for the comparisons made.


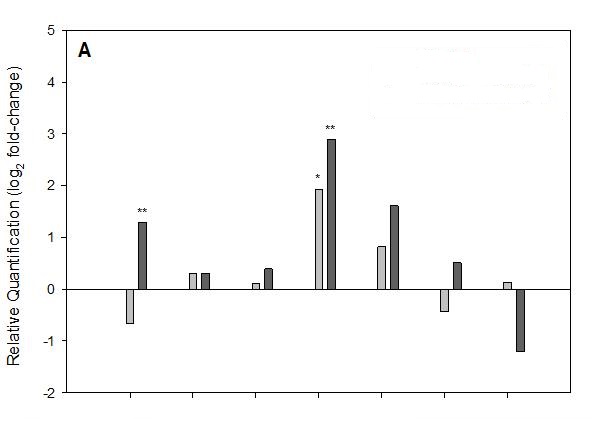

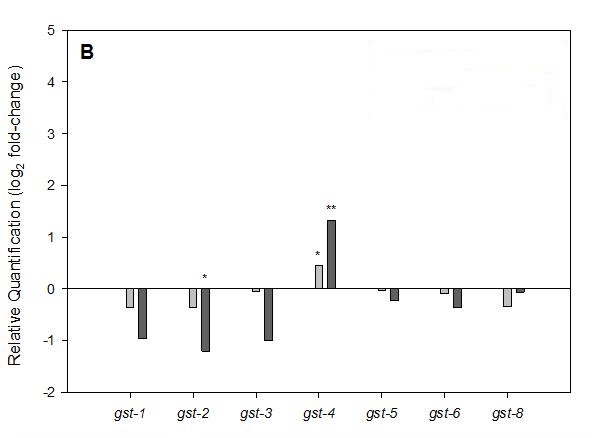


FIGURE S4 Expression levels of genes encoding putative glutathione S-transferases (*gst*) in the reference *Z. tritici* isolate IPO323 (A) and the laboratory mutant L-1.7 (B) after 24 h exposure to their respective fluxapyroxad EC_50_ (light grey) or EC_80_ concentration (dark grey), measured relative to the untreated control using quantitative RT-PCR. Mean of three biological replicates. Statistical significance for each gene is marked by asterisks (*=*p*<0.05 or **=*p*<0.01). See Supplementary Table S5 and S6 for the means of log_2_ fold-change data for the treatments with standard error of the difference for the comparisons made.


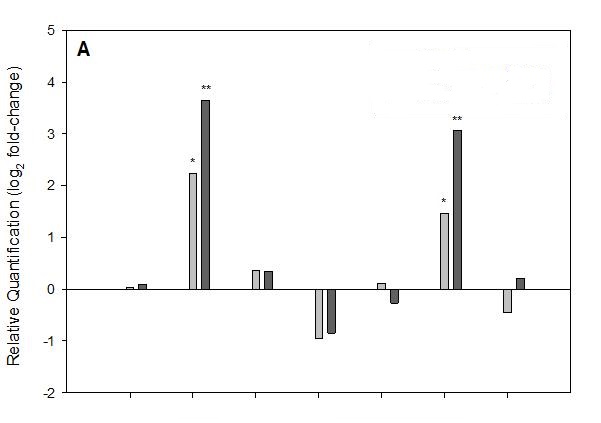

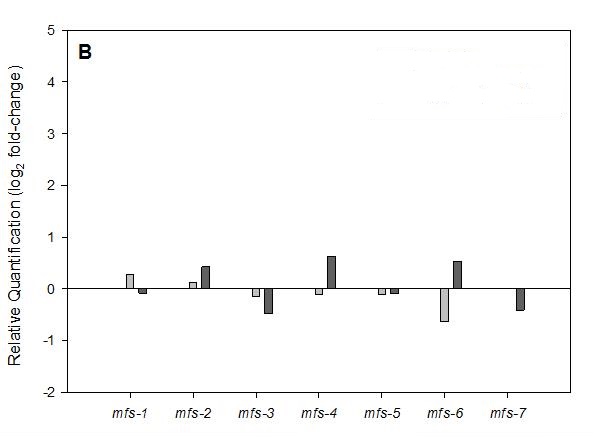


FIGURE S5 Expression levels of genes encoding major facilitator superfamily (*mfs*) drug efflux transport in the reference *Z. tritici* isolate IPO323 (A) and the laboratory mutant L-1.7 (B) after 24 h exposure to their respective fluxapyroxad EC_50_ (light grey) or EC_80_ concentration (dark grey), measured relative to the untreated control using quantitative RT-PCR. Mean of three biological replicates. Statistical significance for each gene is marked by asterisks (*=*p*<0.05 or **=*p*<0.01). See Supplementary Table S5 and S6 for the means of log_2_ fold-change data for the treatments with standard error of the difference for the comparisons made.
